# Physical Activity and Executive Functioning in Children and Adolescents with Congenital Heart Defects: A Scoping Review

**DOI:** 10.3390/jcdd11100309

**Published:** 2024-10-05

**Authors:** Amanda Clifton, Neva Kirk-Sanchez, Gerson Cipriano, James G. Moore, Lawrence P. Cahalin

**Affiliations:** 1 Nicklaus Children’s Hospital, Miami, FL 33155, USA; 2 Department of Physical Therapy, Miller School of Medicine, University of Miami, Coral Gables, FL 33146, USA; nkirksanchez@miami.edu (N.K.-S.); jgmoore@miami.edu (J.G.M.); l.cahalin@miami.edu (L.P.C.); 3 Health Sciences and Technologies Graduate Program, Centro Metropolitano, University of Brasilia (UnB), Conjunto A-Lote 01, Ceilândia, Brasília 72220-900, Brazil; ciprianeft@gmail.com

**Keywords:** children, adolescents, cognition, congenital heart disease, physical activity, exercise, aerobic training

## Abstract

Children and adolescents (C&As) with congenital heart defects (CHDs) have decreased functional capacity and executive functioning (EF) due to brain abnormalities and decreased cerebral perfusion. Exercise may improve EF via increased cognitive demands and cerebral blood supply. The purpose of this review was to identify evidence describing the impact of physical activity (PA) interventions on EF in C&As with CHDs. The following databases were searched from 2000 to 2024: MEDLINE, EMBASE, CINAHL, Scopus, CENTRAL, and PsycInfo. The inclusion criteria consisted of participants aged from birth to 18 years with CHD, interventions related to PA, and EF as an outcome measure. Articles were excluded if adults were included, translation to English was impossible, and full access was unavailable. Of 613 initial articles, 3 were analyzed, with only 1 meeting all inclusion criteria. The included study found significant improvements in self-reported cognitive functioning and parent-reported social functioning after 12 weeks of aerobic exercise in children aged 10–15 years with CHDs. Common themes among the reviewed articles indicated that EF remains impaired throughout the lifespan, children have unique interventional and developmental needs, and research remains limited despite theoretical benefits. Further investigation of the effect of PA on EF in C&As with CHDs is needed.

## 1. Introduction

Children and adolescents (C&As) with congenital heart defects (CHDs) have decreased functional capacity and executive functioning (EF) compared to typical peers. These children often do not have the opportunity or education to participate in safe physical activity (PA) and exercise [1]. CHDs are the most common congenital disability in the United States and impact normal blood flow through the heart and major vessels [2,3]. These defects can result in the need for multiple surgical interventions with diverse timing. Most born with CHDs now survive into adulthood. However, despite increased survival rates due to medical advancements, there continue to be risks, such as hypoxic events during cardiac surgery and impaired cardiorespiratory functioning [4,5,6,7]. Individuals with CHDs face additional challenges due to brain structural abnormalities, chronically decreased oxygenation, and perceived physical limitations [8]. The combination of physical limitation coupled with structural abnormalities of the brain often results in additional impairments in EF.

Executive functioning encompasses skills for goal-directed behavior, which includes self-regulation and three key types of brain function: working memory, mental flexibility, and self-control. These purposeful skills organize behavior and emotional regulation [7]. Children and adolescents with CHDs are at risk of EF impairments due to disease severity, premature birth, and brain injuries, which manifest as changes in brain volume, cortical measurements, and white matter microstructure [8]. Full-term children with cyanotic defects are born with smaller brains and decreased gray matter volumes in the frontal lobe compared to typical peers [7]. Children with single ventricle physiology, such as those with hypoplastic left heart syndrome (HLHS), face greater impairments in EF. Due to staged palliative procedures, such as the Fontan procedure, children with HLHS face prolonged periods of time with reduced oxygenation and perfusion to the brain [7]. This can lead to school-related activity limitations and participation restrictions when engaging in play with peers [6,7,9]. The prefrontal cortex (PFC) is vital for EF and continues to develop into late adolescence [2]. Due to the connection with the developing PFC, EF continues to develop into early adulthood. This progressive maturation has been proposed as the rationale for the favorable effects of dynamic aerobic exercise on EF in C&As with CHDs.

Exercise may improve EF in two ways, including increasing cognitive demands and increasing cerebral blood supply [2]. Cognitive demands during sport-like activities and bimanual coordination stimulate higher thinking processes and require direct attention to the novel and dynamic activity. Additionally, exercise promotes increased cerebral perfusion. In animal studies, aerobic exercise leads to increased blood capillary supply to the PFC and stimulates the growth of new neurons and synapses [10]. In young adults, acute exercise has a positive effect on EF and arousal-related prefrontal activation [11]. Beneficial effects of exercise have been shown during both acute and chronic exercise in C&As [2,11,12,13]. Despite this likely connection, there appears to be a lack of research investigating PA and EF in C&As with CHDs.

A preliminary search of PubMed and Open Science Framework was conducted, and no current or ongoing systematic reviews or scoping reviews on this topic were identified. Due to the lack of published evidence, the authors chose to conduct a scoping review to gain a better understanding of the scope or coverage of the literature related to how participation in PA impacts EF in C&As with CHDs and to identify knowledge gaps related to PA and EF in C&As with CHDs.

## 2. Materials and Methods

The protocol for this scoping review was published in the Open Science Framework (https://doi.org/10.17605/OSF.IO/YFNX3) and created in accordance with the PRISMA extension for scoping reviews (PRISMA-ScR) [14]. An initial search strategy was conducted by one physical therapist to refine search terms and determine the most appropriate databases. The search was then revised by physical therapists with pediatric cardiovascular and pulmonary board specialties and included a total of four physical therapists, two of whom had previous experience conducting scoping reviews. Revisions to this scoping review were made to further refine the search terms and focus on the possible influence of interventions on EF. Article inclusion criteria consisted of participants aged from birth to 18 years with CHD, interventions related to PA (such as resistance training and neurorehabilitation)**,** and EF as an outcome. Articles were excluded if adults were included, translation to English was impossible, and full access was not available. The following databases were searched from 2000 to 2024: MEDLINE, EMBASE, CINAHL, Scopus, CENTRAL, and PsycInfo. An example of a search string for the MEDLINE database is as follows: (Child OR Adolescent OR Pediatric) AND (Neurorehabilitation OR exercise capacity OR physical activity OR resistance training) AND (Congenital heart disease OR congenital heart defects OR Heart Defects OR Congenital surgery) AND (Executive function OR cognitive function OR cognitive performance).

Following the search, all identified citations were collected and exported into Covidence 2022 (Melbourne, Australia), and duplicates were removed. The articles were then independently screened by two team members for eligibility and inclusion criteria. The text words of the titles were analyzed, and the abstracts were reviewed for eligibility based on the inclusion and exclusion criteria. All eligible articles were then retrieved and reviewed. Reasons for the exclusion of sources of evidence in the full text that did not meet inclusion criteria were recorded. A third reviewer was available to resolve any discrepancies in article eligibility and data extraction; however, this was not required. The reference lists of eligible articles were also reviewed for inclusion. Article search terms included children, pediatrics, exercise, PA, CHD, cognition, and EF. The results of the search and the study inclusion process are reported in flow diagram format according to PRISMA-ScR [14,15].

Data were extracted by two team members and placed into a data extraction form, which was adapted from the JBI Manual for Evidence Synthesis [16]. The data extracted included specific details about the participants, study methods, and key findings. Due to the nature of scoping reviews, a formal assessment of methodological quality was not included in this scoping review.

## 3. Results

**Figure 1:**
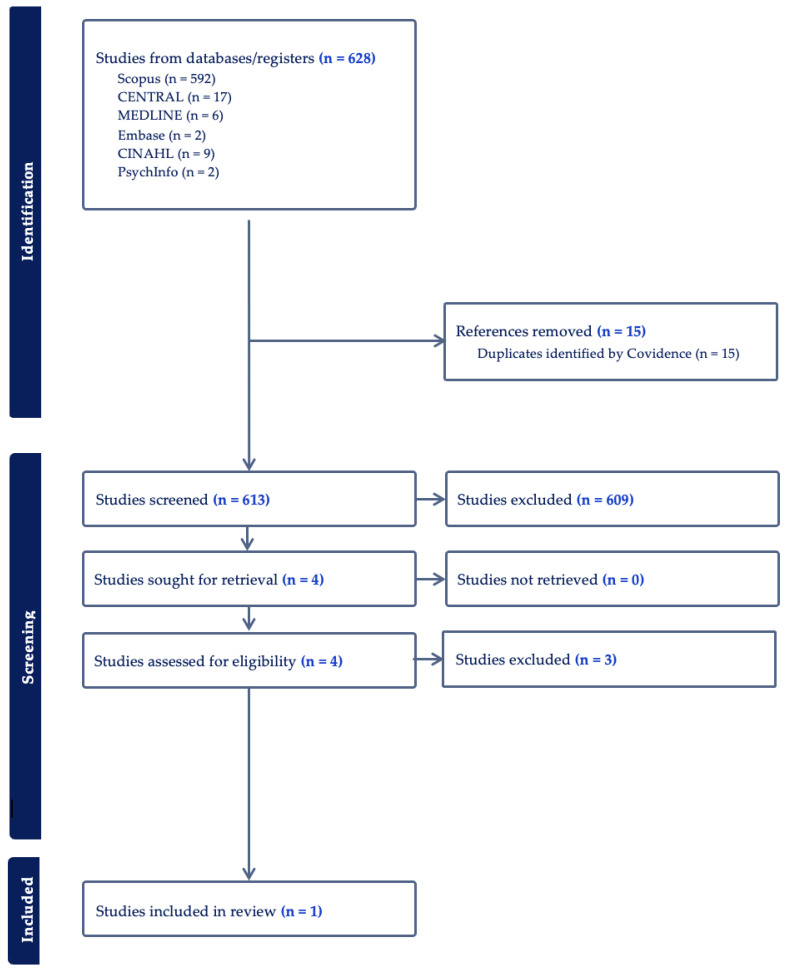
Search strategy flowchart.

As a result of this scoping review, 628 studies were identified, and the findings are reported in Figure 1.

Six studies were duplicates, and one was marked as ineligible. After removal, 424 studies remained available for screening. Articles with titles and abstracts that were not relevant to pediatrics, children with CHD, PA, or EF were excluded. Out of the articles screened, only four abstracts were sought out for full analysis [17,18,19,20]. After a full analysis, one of the four articles examined only included subjects who exceeded our age range inclusion criteria [20], resulting in a total of three articles for further review and possible inclusion [17,18,19]. The data from the three articles are reported in Table 1.

Out of the three articles assessed for eligibility, only the study by Dulfer et al. met all inclusion criteria [17]. Although the study by Dulfer et al. included adults, 46 C&A aged 10–15 years with post-surgical repair for tetralogy of Fallot (ToF) or Fontan circulation underwent a stratified randomization to a control or exercise group. The exercise group (*n* = 25) performed 40 min of aerobic dynamic cardiovascular training preceded and followed by a 10 min warm-up and cooldown. Walking, jogging, running, bicycling, and dynamic play activities were performed three times/week for three months at 60–70% of heart rate reserve. Cognitive functioning was measured using the parent and adult form of the TNO/AZL Child Quality of Life Questionnaire. Significant findings were only found for the 10–15-year cohort of children and included improved self-reported cognitive functioning (*p* < 0.05, *r* = 0.30) and parent-reported social functioning (*p* < 0.05, *r* = 0.30) [17]. It was additionally found that children with lower baseline scores of motor and cognitive functioning had significant improvements after intervention (*z* = −2.54, *p* < 0.05, *r* = 0.57 and *z* = −2.11, *p* < 0.05, *r* = 0.50 respectively) [17].

The article by Cooney et al. was excluded because no intervention was conducted [18]. However, maximal cardiopulmonary exercise tests (CPET) were performed, and EF was assessed by the Wechsler Adult Intelligence Scale (WAIS-IV), the Wechsler Intelligence Scale for Children, 5th edition (WISC-V), and the Tower of London-Drexel (TOL). The study methodology consisted of CPET and neuropsychological testing (NPT) on children with single ventricle heart disease (SVHD) post-Fontan palliation. Exercise testing was completed with a Medgraphics (Saint Paul, MN, USA) metabolic cart. Breath-by-breath data were collected and averaged over 20 s intervals. The subjects performed a symptom-limited test using a ramp protocol on a cycle ergometer. Oxygen saturation, electrocardiogram, and blood pressure were also monitored and measured. A neuropsychologist completed the NPT in a single session on a different day than the CPET. Results from this study found that the percent predicted values for maximal oxygen consumption (VO2 max) and peak heart rate of the SVHD group were substantially lower than values for healthy, age-matched children (44% predicted VO2 max, 76% peak heart). On EF and adaptive functioning, the SVHD cohort scored 1–1.5 standard deviations below normative values. Linear regression analyses found that both VO2 max and anaerobic threshold were associated with parent-rated overall adaptive function. In fact, for one unit increase in VO2max, the global adaptive composite (GAC) increased by 1.13 units (95% CI 0.29, 1.96 *p* = 0.01). An increase in one unit of anaerobic threshold was related to an increase in GAC of 1.61 (95% CI 0.27, 2.94; *p* = 0.02). Finally, peak heart rate was found to be related to sustained visual attention, and an increase by one heartbeat was related to a decrease of 0.4 in the errors of omission T-score (95% CI (−0.71, −0.09); *p* = 0.01). It was further noted that none of the CPET variables were significantly related to working memory, processing speed, EF, or internalizing symptoms, and ventilatory efficiency was not significantly related to any of the NPT variables.

Finally, the article by Verrall et al. was excluded as it was a narrative review without a methodology fitting the inclusion criteria [19]. However, this article provided an important overview of the role exercise may have on EF in children with Fontan physiology. Proposed mechanisms include increasing gray and white matter volumes, enhancing white matter microstructure, and improving functional connectivity. Additionally, Verrall et al. highlighted the effect exercise has on promoting neurogenesis and angiogenesis. The above improvements from PA may improve EF in C&As with CHDs and reduce the risk of neurodegenerative decline and dementia in adults with CHDs, which is a growing concern in this patient population. The Verrall et al. article, along with others noted during the screening process, provides substantial insight into a variety of potential interventions for individuals with CHDs, with exercise being a key method to improve EF in C&As and adults with CHDs [19,21].

## 4. Discussion

During this scoping review process, it was noted that (1) EF was consistently impaired in children with CHD even as they age, (2) interventions for C&As with CHDs need to be comprehensive to meet their unique needs, and (3) research remains limited.

EF is impaired in C&As with CHDs. Infants with CHD are surviving into adulthood, resulting in a focus on improving long-term morbidity and quality of life [21,22]. Known secondary impairments are impaired cognition, behavioral problems, delayed gross and fine motor skills, reduced exercise capacity, and impaired EF [17,18,19,20,21,22,23]. These impairments have been found to be associated with the severity of CHD and were found to begin in infancy and persist throughout adulthood [19,21]. In the Boston Circulatory Arrest Trial, a cohort of patients with dextrotransposition of the great arteries (d-TGA) who underwent the arterial switch operation was followed longitudinally [24,25,26,27]. As infants, these patients had lower than normal scores on the Bayley Scales of Infant Development [24]. As children, abnormal neurodevelopment persisted, and a third had received special services in school [25,26]. At 16 years of age, neurodevelopmental scores continued to be lower than age-matched healthy peers, and abnormal magnetic resonance imaging (MRI) was found [27]. In a cohort of children with Fontan physiology, similar neurodevelopmental outcomes were found, including lower intelligence scores, EF, and an 11% higher frequency of abnormalities on MRI [28]. In the Single Ventricle Reconstruction (SVR) trial, 6-year outcomes found similar results to the aforementioned studies, including deficits of mild and severe motor disabilities occurring at 6 and 11 times the normal incidence rate. [29] Parent-reported EF impairment was also reported to have an odds ratio of 4.37 when compared to typical age and gender-matched peers [30]. As young adults with CHDs, Fox et al. found that one in 10 individuals reported clinically significant difficulties with global EF. These impairments in EF were further associated with health risk behaviors such as increased tobacco use and greater saturated fat intake [20].

An important conclusion from this scoping review is that interventions for C&A swith CHDs need to be comprehensive and tailored to the individual needs of each child or adolescent. In a screened article by Rogers and Dixon, it was found that most pediatric cardiac rehabilitation programs follow the same standards as adult programs, but only a few addressed the unique needs of pediatric patients with CHDs, including concerns for developmental delays, motor skills, and cognitive disruptions [31]. In a study of factors influencing PA in adolescents with complex CHDs, total PA was higher in males, adolescents in formal physical education classes, and those with higher self-efficacy [32]. Additionally, quality-of-life impairments are common, and one in three adults with CHD have mood or anxiety disorders [33]. In view of the above, there is a need for the development and examination of comprehensive rehabilitation programs for C&As with CHDs to improve factors responsible for greater EF.

Research examining the effects of PA on EF is currently limited. Only one published study was found investigating the role that PA interventions may have on EF in children with CHDs [17]. In adults, PA has many well-known cognitive benefits. Research in older adults has found relationships between PA and hippocampal volume preservation as well as increased brain volume [34,35]. In the narrative review by Verrall et al., exercise was proposed as a therapeutic intervention for neurodevelopmental and cognitive dysfunction in people with Fontan circulation [19]. This is a special population of C&A with CHD, as these children face prolonged periods of cyanosis. Proposed mechanisms for potential benefits from PA include improved brain structure and function, neuroprotective and reparative cellular and molecular processes, cognitive reserve, and resilience [19].

It is important to note that cardiorespiratory fitness is associated with gray matter volumes in areas that are typically most sensitive to aging, such as the frontal-parietal and temporal cortices, as well as the hippocampus [35,36]. These areas also have known functions in EF. Although the focus of this study is on C&A with CHD, preliminary research suggests that there is an accelerated age-associated brain volume loss in adults with Fontan circulation [19]. In healthy children, associations between hippocampal volume, fitness, and memory performance have been found [37]. In a study by Riggs et al., aerobic exercises promoted hippocampal growth and white matter recovery in children post-radiation therapy for brain tumors [38]. These same findings may be true in C&A with CHD, for which investigation of the relationship and effects of PA on EF in C&A with CHD are warranted. In the study by Dulfer et al., changes in EF were measured via quality-of-life questionnaires [17]. Future research could include measuring changes via formal cerebral imaging. Additionally, previous research on EF in children with CHD has included performance-based tests of EF, such as the Stroop Test and Trail Making Test [6], as well as reported measures, such as the Behavior Rating Inventory of Executive Function [30]. Including the above outcomes in a comprehensive cardiac rehabilitation program for C&A with CHD is likely to improve the management of C&A with CHD.

This scoping review was limited due to the lack of research meeting the search criteria. Future research is needed to examine if PA interventions impact EF in children with CHD.

## Figures and Tables

**Table 1:** Data extraction form.

Author	Dulfer et al. [17]	Cooney et al. [18]	Verrall et al. [19]
Aims/Purpose	To investigate the effect of an exercise program on HRQoL in children and adolescents with TOF or Fontan circulation.	To characterize the relationship between neurodevelopment and exercise capacity in SVHD post-Fontan by evaluating associations between CPET and clinical NPT.	To discuss current interventions and evidence supporting exercise as a potential intervention for improving cognitive functioning in people with Fontan circulation.
Population	Ninety-three participants, ages 10–25 years, had a surgical repair for ToF or with Fontan circulation.	Twenty-three participants, ages 7–17 years old, with Fontan circulation.	Discusses impact from fetus to aging adult.
Methodology	Stratified, randomized controlled intervention conducted in five pediatric centers in The Netherlands. Random allocation with a ratio of 2:1 in a 12-week period with an exercise program of three times per week or a control group.	Retrospective, cross-sectional pilot study conducted in the United States. One-time conduction of CPET with gas analysis and one-time conduction of NPT.	Narrative summary and discussion with the following categories: established neurodevelopmental and cognitive interventions; exercise, cognition, and Fontan physiology; neural mechanisms underpinning the exercise–cognition relationship; and psychosocial and behavioral mediators of the exercise–cognition relationship.
Interventions	Exercise program consisted of three one-hour long training sessions a week. Patients already active were encouraged to continue to perform activities two times a week. An hour session consisted of 10 min warm up, 40 min aerobic training, and 10 min cooldown. Participants trained within given heart rate ranges.	Exercise testing was performed with a Medgraphics (Saint Paul, MN, USA) metabolic cart. Breath-by-breath data were collected and averaged over 20 s intervals. Patients performed a symptom-limited test using a ramp protocol on a cycle ergometer. Oxygen saturation was monitored, and electrocardiogram and blood pressure were also measured. NPT was conducted in a single session on a day separate from the CPET. Standardized scores were assessed from tests of working memory, processing speed, sustained visual attention, executive function, parent ratings of adaptive function, and internalizing problems. Scores were standardized by age.	Individuals with Fontan physiology have a greater risk of neurodevelopmental and cognitive impairments. Exercise is low-risk and has benefits on physical and cognitive functioning. Future research is needed to provide exercise prescriptions and determine accessible interventions.
Outcomes	At baseline and follow-up after 12 weeks, participants and parents, as appropriate, completed the HRQoL measures: for the 10–15 group, TACQOL CF and TACQOL PF; for the 16–25 group, SF-36 and CONHD-TAAQOL; and for the total group 10–25, LAS.	CPET measures: VO2max indexed to body weight, anaerobic threshold, peak heart rate, ventilatory efficiency, pulmonary vasodilator use, and RER. Results were compared to percent predicted values for VO2max and peak heart rate based on gender, height, and weight. NPT measures: executive function (WAIS-IV or WISC-V and TOL), attention (CPT-2,3), adaptive function (ABAS-2,3), and emotional function (BASC-2,3).	
Key Findings	Compared with the control group, children aged 10–15 years in the exercise group improved significantly in self-reported cognitive functioning and parent-reported social functioning. Increase was noted in this group with lower baseline HRQoL. Participants aged 16–25 years did not change their HRQoL.	Higher VO2max and anaerobic thresholds were related to better adaptive functioning scores, and higher peak heart rates were related to better scores when measuring sustained visual attention. The relationship appeared strongest in relation to adaptive function, as both higher VO2 max and anaerobic threshold were significantly associated with a higher global adaptive composite score. CPET variables related to working memory, processing speed, executive functioning, or internalizing symptoms were not significant. Ventilatory efficiency was not significantly related to any of the NPT variables.	Interventions for impaired neurodevelopment and cognitive dysfunction in people with Fontan circulation are lacking.

HRQoL = health-related quality of life; ToF = tetralogy of Fallot; TACQOL CF and PF = TNO/AZL Child Quality of Life Questionnaire Child Form and Parent Form; SF-36 = Short Form-36 Health Survey; CONHD-TAAQOL = Congenital Heart Disease-TNO/AZL Adult Quality of Life Questionnaire; LAS = linear analog scale; SVHD = single ventricle heart disease; CPET = cardiopulmonary exercise testing; NPT: neuropsychological testing; VO2max = maximal oxygen consumption; RER = respiratory exchange ratio; WAIS-IV = Wechsler Adult Intelligence Scale; WISC-V = Wechsler Intelligence Scale for Children, 5th edition; TOL = Tower of London-Drexel; CPT-2,3= Conners’ Continuous Performance Task, versions 2 and 3; ABAS-2,3 = Adaptive Behavior Assessment System, Second and Third editions; and BASC-2,3 = Behavior Assessment System for Children, versions 2 and 3.

## Data Availability

Not applicable.
